# Medical Association

**Published:** 1849-04

**Authors:** 


### MEDICAL ASSOCIATION.

The profession will bear in mind that the American Medical Association meets in Boston on the first Thursday in May. Medical Societies, Colleges, etc., it is to be hoped, will appoint delegates, and in selecting them will fix upon such as will attend the meeting. The Association will lose the character of national which it is so desirable to have maintained if members are not present at its meetings from all quarters of the union. We trust that the approaching assemblage will be the largest yet seen, and can venture to promise those who attend that they will be hospitably received by their brethren in Boston, and will find the meeting full of intellectual and social interest.

